# Promiscuous involvement of metabotropic glutamate receptors in the storage of *N*-methyl-d-aspartate receptor-dependent short-term potentiation

**DOI:** 10.1098/rstb.2023.0445

**Published:** 2024-07-29

**Authors:** Rachael Ingram, Arturas Volianskis

**Affiliations:** ^1^ Centre for Neuroscience, Surgery and Trauma, Blizard Institute, Barts and The London School of Medicine and Dentistry, Queen Mary University of London, London E1 2AT, UK; ^2^ School of Biosciences, Cardiff University, Museum Avenue, Cardiff CF10 3AX, UK

**Keywords:** short-term potentiation, long-term potentiation, NMDA receptor, metabotropic glutamate receptor, synaptic plasticity, learning and memory

## Abstract

Short- and long-term forms of *N*-methyl-d-aspartate receptor (NMDAR)-dependent potentiation (most commonly termed short-term potentiation (STP) and long-term potentiation (LTP)) are co-induced in hippocampal slices by theta-burst stimulation, which mimics naturally occurring patterns of neuronal activity. While NMDAR-dependent LTP (NMDAR-LTP) is said to be the cellular correlate of long-term memory storage, NMDAR-dependent STP (NMDAR-STP) is thought to underlie the encoding of shorter-lasting memories. The mechanisms of NMDAR-LTP have been researched much more extensively than those of NMDAR-STP, which is characterized by its extreme stimulation dependence. Thus, in the absence of low-frequency test stimulation, which is used to test the magnitude of potentiation, NMDAR-STP does not decline until the stimulation is resumed. NMDAR-STP represents, therefore, an inverse variant of Hebbian synaptic plasticity, illustrating that inactive synapses can retain their strength unchanged until they become active again. The mechanisms, by which NMDAR-STP is stored in synapses without a decrement, are unknown and we report here that activation of metabotropic glutamate receptors may be critical in maintaining the potentiated state of synaptic transmission.

This article is part of a discussion meeting issue ‘Long-term potentiation: 50 years on’.

## 1. Introduction

High-frequency activation of excitatory glutamatergic synapses in the adult hippocampus induces two forms of *N*-methyl-d-aspartate receptor (NMDAR)-dependent long-lasting potentiation [1–4], which are most frequently referred to as short-term potentiation (STP) and long-term potentiation (LTP). NMDAR-dependent STP is often seen as the initial, transient, declining phase of potentiation that leads to a stable phase of potentiation, sustained NMDAR-dependent LTP [4]. Inductions of STP and LTP differ in terms of their sensitivity to a variety of NMDAR antagonists [5–8] and second messenger inhibitors [9–12]. STP and LTP also differ in terms of second messenger involvement in their expression [13–15]; they are pharmacologically and physiologically distinct [16–18].

NMDAR-dependent STP is sometimes confused with NMDAR-independent types of synaptic plasticity (e.g. paired-pulse facilitation, frequency facilitation and post-tetanic potentiation (PTP)) that are collectively known under the umbrella term of ‘short-term plasticity’, sharing the acronym of STP (see [17,19] and also this issue [20] for further discussion of the terminology). Due to such confusion, NMDAR-dependent STP has been termed transient LTP (transient phase of LTP or t-LTP [4]) in contrast to the stable or sustained LTP (s-LTP), which is less prone to decline [21,22]. Indeed, because of its NMDAR-receptor dependence and long-lasting duration, NMDAR-dependent STP is more akin to NMDAR-dependent LTP than to the various forms of NMDAR-independent short-term plasticity. However, despite its faults, it has proved near to impossible to change the established nomenclature [23] that has been adopted and used by the field for many years. Therefore, in order to avoid any further ambiguity in the use of the acronyms, throughout the rest of this paper we will be referring to NMDAR-dependent STP as NMDAR-STP. We will be referring to NMDAR-dependent LTP simply as LTP.

During the past 50 years, LTP [1] has established itself as a reputable correlate of long-lasting memory storage [23–27] while the idea that NMDAR-STP could be the synaptic mechanism behind the encoding of shorter-lasting memories [28] has been slowly gaining traction over the years [4,6,12,18,29,30]. Notably, the longevity of both NMDAR-STP and LTP is not absolute but depends on the reference frame and actions of the observer, whose measurement of NMDAR-STP and LTP introduces uncertainty in the outcome of the experiments by affecting the duration of synaptic plasticity [4,31].

NMDAR-STP and NMDAR-LTP are frequently co-induced and co-expressed in adult hippocampal glutamatergic synapses *in vitro* [4] and *in vivo* [2]. During experiments in hippocampal slice preparations, potentiation of synaptic responses is probed by using low-frequency presynaptic stimulation (e.g. 0.017–0.13 Hz), which retains the stability of baseline postsynaptic responses. After the induction of potentiation, the sensitivity of the responses to synaptic stimulation changes, and the decay of NMDAR-STP—or its depotentiation—happens readily when probed with baseline stimulation frequencies that do not affect LTP. Thus, in experiments using continuous low-frequency test stimulation NMDAR-STP decays to a stable level of LTP in about an hour [4]. The depotentiation of LTP requires higher frequencies of stimulation than NMDAR-STP (e.g. 1–2 Hz [31–34]). At baseline stimulation frequencies *in vitro,* LTP can last without a decrement for hours [35], while LTP *in vivo* has been shown to last for days [36,37], months [38] and even years [22].

The duration of NMDAR-STP can be controlled by changing the frequency of the depotentiating stimulation [2,4,12,39]. NMDAR-STP declines faster in experiments with more frequent pre-synaptic stimulation than in experiments with slower stimulation [2,4,39]. The process of the NMDAR-STP decay can be suspended by temporally pausing the afferent stimulation [4,5,12,18], for a variety of time periods and up to 6 h *in vitro* [4]. In such experiments, the levels of NMDAR-STP are stored in synapses during periods of synaptic inactivity and NMDAR-STP does not decline until the stimulation is resumed, and synapses are re-activated. Such use-dependent storage of potentiation can temporarily increase the dominance of some synaptic connections over others, permitting the formation of dynamic cell assembles and short-term memories. Reactivation of the set cell assemblies, which have been left in a potentiated state by a momentary experience, would then be central during recall and in cognition [28]. As suggested by Donald Hebb, such synaptic memory processes would be able to account for memory types that cannot be explained by either reverberatory activity or by a structural change [40]. Indeed, it has been now shown that during cognition humans can hold working memory information using activity-silent synaptic mechanisms [41], relying possibly on NMDAR-STP-like processes.

At present, the mechanisms underlying the synaptic storage of NMDAR-STP are largely unknown except that it has been observed that NMDA receptors can be involved in regulating the decay of NMDAR-STP, after its initial induction [4]. The involvement of metabotropic glutamate receptors (mGluRs) in the storage of NMDAR-STP has not been investigated previously and we have tested here whether or not a specific mGluR is involved in mediating the storage of NMDAR-STP.

## 2. Material and methods

**Figure 1.**
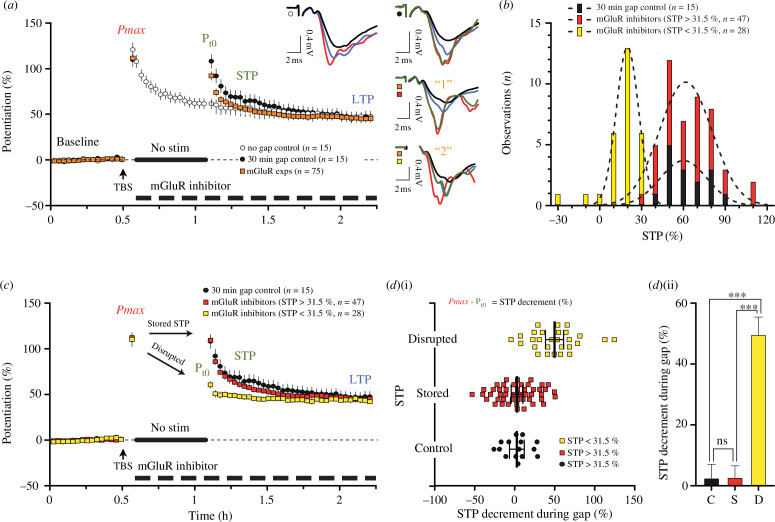
Involvement of mGluRs in the storage of NMDAR-STP. (*a*) TBS (timing indicated by the arrow) was used to induce NMDAR-STP and LTP of f-EPSPs after recording of stable baseline responses (Baseline; black waveforms) in three groups of experiments (experiment numbers are displayed on the panel). In the first group of control experiments (white circles; no gap control), NMDAR-STP was depotentiated by stimulation (1/15 s, 0.067 Hz) immediately after the recording of the maximal levels of NMDAR-dependent potentiation (*P*_max_; red waveform) to a stable level of LTP (blue waveform). In the second group of control experiments (black circles, 30 min gap control) NMDAR-STP was stored for 30 min by a delay (gap) in stimulation (indicated by the thick black line; no stim). After the resumption of the stimulation (*P*_*t*0_; green waveform) NMDAR-STP was depotentiated to a stable level of LTP (blue waveform). In the third group of experiments (orange squares; mGluR exps) an mGluR inhibitor was applied after the recording of *P*_max_, during the 30 min gap in stimulation and throughout the rest of the experiments (indicated by the dashed line). In this group of experiments, NMDAR-STP could be either stored as in controls (outcome ‘1’, green f-EPSP (*P*_*t*0_) overlapping the red, *P*_max_) or not stored (outcome ‘2’, green f-EPSP (*P*_*t*0_) overlapping the blue, LTP). (*b*) Frequency distributions of NMDAR-STP amplitudes (10% bins) after the resumption of the stimulation for controls (black) and for experiments using mGluR inhibitors in which NMDAR-STP was either disrupted (yellow, NMDAR-STP < 31.5%) or stored successfully (red; NMDAR-STP > 31.5%) were fitted with Gaussian curves (dashed lines). When comparing the Gaussian curves, the mean amplitude and *σ* of the disrupted NMDAR-STP distribution (yellow, 19.9 ± 8.0%) were significantly smaller than those in control distribution (black, 60.1 ± 16.8%, *p* < 0.0001), or in experiments with successful storage (red distribution, 61.6 ± 18.8%, *p* < 0.0001). (*c*) The datasets from (*b*) are shown plotted as mean values of potentiation ± s.e.m., over time. After the resumption of stimulation, the mean NMDAR-STP amplitude in the stored NMDAR-STP group (red squares) was 64.2 ± 2.6% while NMDAR-STP in the disrupted group (15.8 ± 2.4%) was much smaller. The values of *P*_max_ and LTP in both groups were consistent with those in the control (black circles). A large decrement in potentiation during the 30 min gap in stimulation (*P*_max_ versus *P*_*t*0_) was seen in the disrupted NMDAR-STP group but not in the stored. (*d)(*i) Decrement in NMDAR-STP during the 30 min gap in stimulation, individual experiment data from the datasets in (*b*) (*P*_max_ − *P*_*t*0_; mean values ± confidence intervals are also displayed). (*d*)(ii) The mean decrement in NMDAR-STP ± s.e.m. (data from (*d*)(i) during the gap in stimulation: the control (C, black), the stored mGluR group (S, red) and the disrupted NMDAR-STP group (D, yellow). Ordinary one-way ANOVA *F*_2,87_ = 33.09; *p* < 0.001, significant differences after Bonferroni’s MCT.

Experiments were performed as described previously in detail [4,5], after institutional approval and according to national and European Union guidelines for animal care, using Schedule 1 procedures for tissue preparation (the UK Scientific Procedures Act, 1986). Briefly, dorsal hippocampal slices were prepared from adult Wistar rats (*n* = 76, 220–300 g, Charles River Labs UK or Envigo UK), after isoflurane anaesthesia and cervical dislocation. Field excitatory postsynaptic potentials (f-EPSPs) were recorded under submerged recording conditions, from the CA1b area of the Schaffer collaterals, which were stimulated at the border between CA3 and CA1 (0.067 Hz, detailed methods in [4]). The slopes of the f-EPSPs were measured and results are presented as mean ± s.e.m. (% potentiation over baseline), plotted over time at 2 min intervals (figure 1). Potentiation was induced by theta-burst stimulation (TBS, four pulses at 100 Hz repeated 10 times at a 5 Hz frequency [42,43]) that was applied after the 30 min recording of stable baseline responses. The baseline stimulation was then stopped for 3 min to avoid contamination of the estimate of maximal NMDAR-dependent potentiation (*P*_max_) by PTP, which is NMDAR independent [4]. Following the recording of *P*_max_, NMDAR-STP was either allowed to decay in response to stimulation or the stimulation was discontinued for 30 min and NMDAR-STP was then seen after the resumption of the stimulation decaying to a stable level of LTP. Exponential fitting was used to determine the decay times (*τ*) of NMDAR-STP. The measurements of *P*_max_, the level of potentiation after the resumption of stimulation (potentiation at time zero, *P*_*t*0_), and LTP report % increase over the baseline of the f-EPSP slope, while the amplitude of NMDAR-STP (%) is the difference between *P*_*t*0_ and LTP [4].

During the pharmacological experiments, mGluR antagonists were applied after the recording of *P*_max_, during the 30 min delay in stimulation and kept throughout the experiment. The ligands included a variety of competitive and allosteric compounds: mGluR antagonists LY367385 (mGluR1-preferring, group I antagonist), LY341495 (mGluR2-preferring, group II/III antagonist) and subtype-selective allosteric inhibitors targeting mGluR1 (YM298198), mGluR5 (MTEP) and mGluR7 inhibitor (XAP044). Compounds were purchased from either ABCAM Biochemicals UK or Tocris Bioscience UK, prepared as stock solutions according to the manufacturer’s guidelines, stored frozen and added to experimental solutions when needed.

Experiments were performed in a randomized manner with control and pharmacological experiments interleaved. Paired Student’s *t*-tests were used for the within-group comparisons, unpaired *t*-tests were used to compare between different groups and frequency distributions were compared using *F*-test (GraphPad Prism). One-way ANOVA followed by Bonferroni’s multiple comparison test (MCT) was used to compare mean values of more than two groups (GraphPad Prism).

## 3. Results

### (a) Stochastic involvement of metabotropic glutamate receptors in the storage of *N*-methyl-d-aspartate receptor-dependent short-term potentiation

Two types of control experiments (figure 1*a*, white circles versus black circles) were interleaved with the pharmacological experiments, which used the mGluR antagonists (figure 1*a*, orange squares). In both types of the controls, TBS was applied after the 30 min recording of stable baseline responses and maximal levels of NMDAR-dependent potentiation (*P*_max_) were estimated after a 3 min delay to avoid NMDAR-independent PTP [4,44]. Following the recording of *P*_max_, NMDAR-STP was either allowed to decay in response to stimulation (white circles, *P*_max_ = 120.7 ± 7.6%, NMDAR-STP = 61.9 ± 5.3%, *τ* = 22.8 ± 3.9 min, LTP = 45.8 ± 3.7%, *n* = 15) or the stimulation was discontinued for 30 min (indicated by the thick line, black circles *n* = 15, figure 1*a*). After the resumption of the stimulation NMDAR-STP was seen decreasing to a stable level of LTP, similar to the experiments without the delay in stimulation (black circles versus white circles respectively, figure 1*a*). In the experiments with the 30 min gap in stimulation, there was virtually no decrement in the levels of potentiation during the pause (*P*_max_ − *P*_*t*0_ = 2.7 ± 4.3%), when compared between the *P*_max_ (109.8 ± 7.8% over baseline, *n* = 15) and the level of potentiation 2 min after the resumption of stimulation (potentiation at time zero, *P*_*t*0_ = 107.1 ± 6.6%, *p* = 0.53). After the resumption of the stimulation NMDAR-STP (62.4 ± 4.0%) decayed with a time constant of 15.7 ± 2.5 min, to a 44.7 ± 4.8% level of LTP above baseline (black circles, figure 1*a*).

Pharmacological experiments (orange squares, figure 1*a*, *n* = 75) using the mGluR antagonists were performed in the same way as the control experiments with the 30 min pause in stimulation (black circles, figure 1*a*) except that ligands were bath-applied after the recording of the *P*_max_, and then kept throughout (dashed bar, figure 1*a*). In these experiments, there was a significant decrement (20.5 ± 4.0%) in the levels of potentiation when compared between the *P*_max_ and *P*_*t*0_ (111.5 ± 3.6% versus 91.1 ± 3.6%, respectively, *p* < 0.00001). NMDAR-STP (46.1 ± 3.3%) declined faster than in the control (*τ* = 8.4 ± 0.8 min versus 15.7 ± 2.5 min, respectively, *p* = 0.0007) to the same level of LTP as in the control (45.0 ± 1.6% versus 44.7 ± 4.8%, respectively, *p* = 0.95).

Examination of the pharmacological dataset revealed a large number of single experiments that were virtually NMDAR-STP lacking (see the waveforms in figure 1). Concurrently, many of the other experiments seemed to be similar to the controls and we sought a strategy to sort the results in an unbiased way. As reported above, the amplitude of NMDAR-STP in the control experiments with the 30 min gap in stimulation was 62.4% and *σ* was 15.4%. All the single experiments, in both the control and the pharmacological datasets, were therefore sorted according to the magnitude of NMDAR-STP after the resumption of the stimulation by using the empirical 95% rule for separating independent Gaussian distributions (i.e. 62.4% − 2*σ; NMDAR-STP < 31.5% and NMDAR-STP > 31.5%). Notably, the control dataset did not contain any experiments in which NMDAR-STP was smaller than 31.5% (range 39.9–93.2%, black bars, figure 1*b*) while 28 out of 75 pharmacological experiments showed NMDAR-STPs that were smaller than 31.5% (range −29.2% to 30.4%, yellow bars, figure 1*b*). The residual 47 experiments, in which NMDAR-STP was larger than 31.5%, are shown plotted in red (range 32.4–112.3%, figure 1*b*). The datasets were fitted with Gaussian distributions demonstrating that the ‘large’ NMDAR-STP group (NMDAR-STP > 31.5%, red bars, figure 1*b*) was virtually indistinguishable from the control group in terms of its mean NMDAR-STP values and *σ* (*F*_2,12_ = 0.0492, *p* = 0.9522). The mean NMDAR-STP values and *σ* in the ‘small’ NMDAR-STP group (NMDAR-STP < 31.5%) were statistically different from those in both the ‘large’ NMDAR-STP group and the control experiments (*F*_4,36_ = 49.38, *p* < 0.0001).

The datasets that emerged through the sorting procedure revealed that the mean *P*_max_ values were very similar when compared between the ‘small’ and ‘large’ NMDAR-STP groups (110.3 ± 5.9% versus 112.3 ± 4.7%, respectively, *p* = 0.79, yellow squares versus red squares, figure 1*c*). The levels of LTP were also similar between the two datasets (44.7 ± 2.3% versus 45.2 ± 2.3%, *p* = 0.88). However, storage of NMDAR-STP was clearly disrupted in the ‘small’ NMDAR-STP group that showed a 50% decrement in potentiation when compared between the *P*_max_ and the *P*_*t*0_ (*P*_max_ − *P*_*t*0_ = 49.9 ± 5.5%, *p* < 0.0001). The storage of NMDAR-STP was unaffected in the ‘large’ NMDAR-STP group (*P*_max_ − *P*_*t*0_ = 2.9 ± 3.7%, *p* = 0.43). The amount of decrement in potentiation during the 30 min period without stimulation differed significantly when compared between the control and the ‘small’ NMDAR-STP group (figure 1*d*(i),(ii), *p* < 0.0001, Bonferroni’s MCT, black versus yellow), while there was no difference when compared between the control and the large NMDAR-STP group (figure 1*d*(i),(ii), *p* > 0.99, Bonferroni’s MCT, black versus red). We were interested whether the observations of stored and disrupted NMDAR-STP were stemming from particular animals or could be attributed to the application of mGluR antagonists. Experiments in slices from the same animals showed incidences of both stored and disrupted NMDAR-STP (table 1) and we concluded that application of mGluR antagonists can disrupt NMDAR-STP in an unpredictable fashion.

**Table 1.** Incidence of stored and disrupted NMDAR-STP across the animals used. (Sixty-one rats were used in the experiments (exps, *n* refers to animal numbers) with the 30 min pause in stimulation and multiple-slice recordings were performed in slices from 24 animals. In terms of the storage and disruption of NMDAR-STP a variety of outcomes were observed, showing no association with the animal identity (n/o means not observed). Thus, in some of the animals either only stored or only disrupted NMDAR-STPs were observed (100% stored STP; 100% disrupted STP) while in some other animals both stored and disrupted STPs were prevalent. The experiments were performed over a two-year period and the observations were randomly spread out over the time.)

number of animals	100% stored STP	100% disrupted STP	stored and disrupted
1-slice exps (*n* = 37)	*n* = 25 (67.6%)^a^	*n* = 12 (32.4%)	—
2-slice exps (*n* = 19)	*n* = 10 (52.6%)	*n* = 3 (15.8%)	*n* = 6 (31.6%)
3-slice exps (*n* = 5)	*n* = 3 (60.0%)	n/o (0%)	*n* = 2 (40.0%)^b^

^a^ Includes six controls without application of antagonists.

^b^ Two slices stored and one disrupted.

### (b) Involvement of multiple metabotropic glutamate receptors in the storage of *N*-methyl-d-aspartate receptor-dependent short-term potentiation

**Figure 2.**
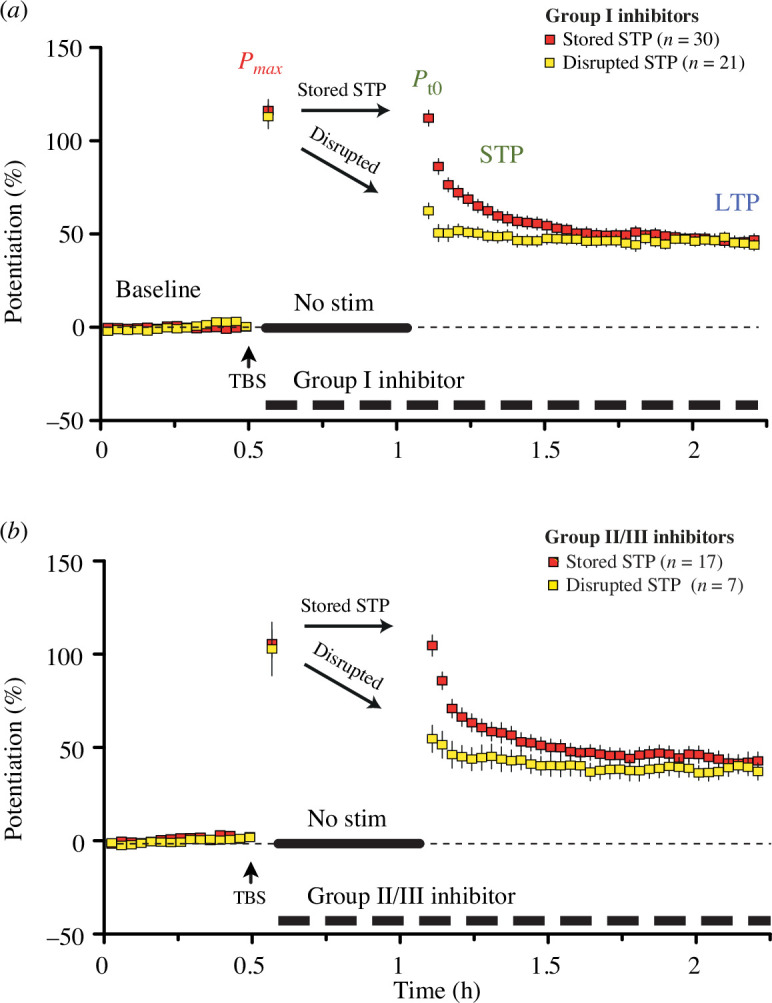
Group I and group II/III inhibitors cause disruption of NMDAR-STP. (*a*) Application of group I mGluR antagonists during the gap in stimulation disrupted storage of NMDAR-STP in 21 out of 51 experiments (42.2%). The stored NMDAR-STP group (*P*_max_ − *P*_*t*0_ = 4.0 ± 4.8%, *p* = 0.4): *P*_max_ = 116.1 ± 6.0%, *P*_*t*0_ = 112.0 ± 4.2%, NMDAR-STP = 65.3 ± 3.1%, *τ* = 9.3 ± 1.0 min, LTP = 46.8 ± 2.9%. The disrupted NMDAR-STP group (*P*_max_ − *P*_*t*0_ = 50.5 ± 6.8%, *p* < 0.00001): *P*_max_ = 112.8 ± 6.4%, *P*_*t*0_ = 62.4 ± 3.4%, NMDAR-STP = 15.9 ± 3.1%, LTP = 46.4 ± 2.6%. In this and subsequent figures we are not reporting *τ* values of the disrupted NMDAR-STP groups owing to the ambiguity of measurements. (*b*) Application of group II/III mGluR antagonists also disrupted the storage of NMDAR-STP (7/24, 29.1%). The stored NMDAR-STP group (*P*_max_ − *P*_*t*0_ = 1.0 ± 5.8%, *p* = 0.87): *P*_max_ = 105.6 ± 7.4%, *P*_*t*0_ = 104.6 ± 5.5%, NMDAR-STP = 62.3 ± 4.6%, *τ* = 10.2 ± 1.3 min, LTP = 42.3 ± 3.6%. The disrupted NMDAR-STP group (*P*_max_ − *P*_*t*0_ = 48.2 ± 9.8%, *p* < 0.003): *P*_max_ = 102.8 ± 14.4%, *P*_*t*0_ = 54.7 ± 7.1%, NMDAR-STP = 15.3 ± 3.4%, LTP = 39.4 ± 4.6%.

As described above, a significant proportion of the pharmacological experiments (37%) showed an almost complete disappearance of NMDAR-STP when mGluR receptor inhibitors were applied during the pause in stimulation. Surprisingly, disrupted NMDAR-STP experiments were seen with both group I (41%, *n* = 51; figure 2*a*) and group II/III mGluR inhibitors (29%, *n* = 24; figure 2*b*). In all of these experiments, the levels of *P*_max_ and LTP remained consistent with those in the controls, suggesting an NMDAR-STP-specific effect of the antagonists.

**Figure 3.**
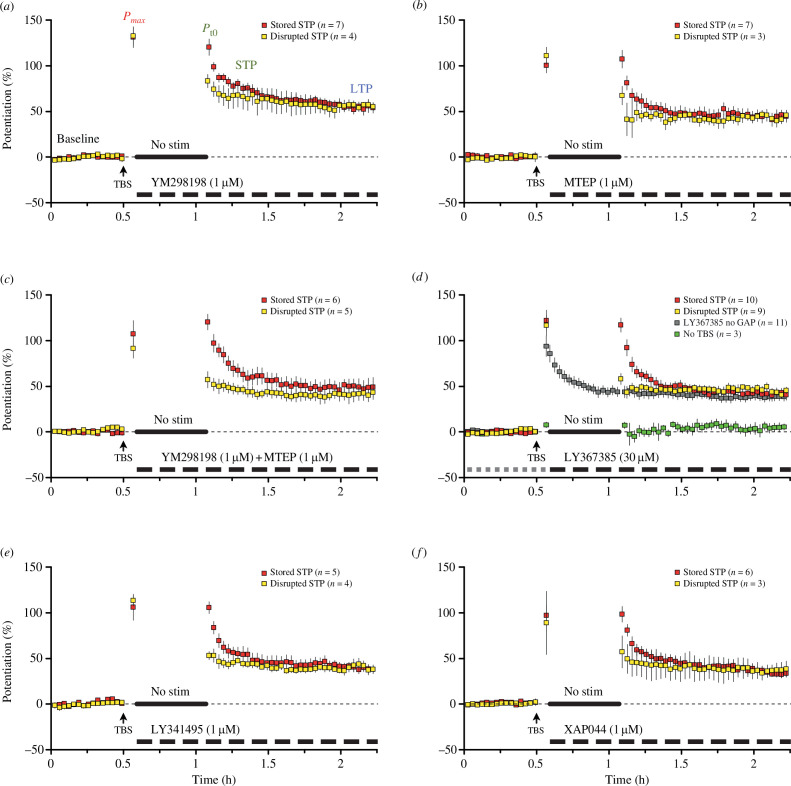
No receptor specificity for the mGluR-mediated disruption of NMDAR-STP. (*a*) Application of the mGluR1 antagonist YM298198 disrupted storage of NMDAR-STP in 4 out of 11 experiments (36.4%). The stored NMDAR-STP group (*P*_max_ − *P*_*t*0_ = 11.2 ± 5.7%, *p* = 0.1): *P*_max_ = 131.4 ± 11.3%, *P*_*t*0_ = 120.2 ± 8.3%, NMDAR-STP = 64.9 ± 8.7%, *τ* = 13.5 ± 1.9 min, LTP = 55.3 ± 4.1%. The disrupted NMDAR-STP group (*P*_max_ − *P*_*t*0_ = 52.6 ± 7.9%, *p* = 0.007): *P*_max_ = 132.7 ± 9.2%, *P*_*t*0_ = 80.0 ± 7.5%, NMDAR-STP = 22.1 ± 2.6%, LTP = 57.9 ± 7.7%. (*b*) mGluR5 antagonist MTEP disrupted the storage of NMDAR-STP in 3 out of 10 experiments (30%). The stored NMDAR-STP group (*P*_max_ − *P*_*t*0_ = −6.8 ± 9.6%, *p* = 0.5): *P*_max_ = 100.4 ± 8.3%, *P*_*t*0_ = 107.2 ± 9.6%, NMDAR-STP = 63.0 ± 7.4%, *τ* = 6.7 ± 1.3 min, LTP = 44.2 ± 5.9%. The disrupted NMDAR-STP group (*P*_max_ − *P*_*t*0_ = 43.6 ± 9.8%, *p* = 0.047): *P*_max_ = 110.8 ± 9.3%, *P*_*t*0_ = 67.2 ± 2.8%, NMDAR-STP = 23.9 ± 0.7%, LTP = 43.3 ± 3.4%. (*c*) Co-application of YM298198 (YM) and MTEP disrupted storage of NMDAR-STP 45% of experiments (5 out of 11). The stored NMDAR-STP group (*P*_max_ − *P*_*t*0_ = −6.0 ± 12.8%, *p* = 0.7): *P*_max_ = 107.0 ± 14.7%, *P*_*t*0_ = 113.0 ± 8.0%, NMDAR-STP = 66.9 ± 4.8%, *τ* = 11.2 ± 2.8 min, LTP = 46.2 ± 8.1%. The disrupted NMDAR-STP group (*P*_max_ − *P*_*t*0_ = 38.1 ± 9.9%, *p* = 0.02): *P*_max_ = 91.4 ± 10.8%, *P*_*t*0_ = 53.3 ± 6.3%, NMDAR-STP = 12.4 ± 6.6%, LTP = 41.0 ± 4.6%. (*d*) Application of mGluR1 preferring antagonist LY367385 disrupted storage of NMDAR-STP in 9 out of 19 experiments (47%). The stored NMDAR-STP group (*P*_max_ − *P*_*t*0_ = 12.6 ± 8.9%, *p* = 0.2): *P*_max_ = 121.8 ± 11.6%, *P*_*t*0_ = 109.1 ± 7.9%, NMDAR-STP = 66.1 ± 4.8%, *τ* = 6.9 ± 1.5 min, LTP = 43.0 ± 5.2%. The disrupted NMDAR-STP group (*P*_max_ − *P*_*t*0_ = 58.6 ± 14.1%, *p* = 0.003): *P*_max_ = 116.5 ± 11.4%, *P*_*t*0_ = 57.9 ± 4.6%, NMDAR-STP = 12.5 ± 5.9%, LTP = 45.4 ± 3.3%. In experiments without a gap, LY367385 did not affect the induction of plasticity and did not disrupt the decay of NMDAR-STP, when applied prior to TBS (grey dashed bar) and kept throughout (grey squares); *P*_max_ 93.9 ± 11.3%, NMDAR-STP 51.2 ± 5.8%, *τ* = 11.0 ± 1.1 min, LTP 37.8 ± 3.7%. In gap experiments without TBS, LY367385 did not disrupt baseline transmission (green squares, *P*_max_ − *P*_*t*0_ = 1.4 ± 3.7%, *p* = 0.7). (*e*) Application of 1 μM group II (mGluR 2 and 3) and group III (mGluRs 4, 6, 7 and 8) antagonist LY341495 disrupted storage of NMDAR-STP in 4 out of 9 experiments (44%). The stored NMDAR-STP group (*P*_max_ − *P*_*t*0_ = 0.6 ± 11.2%, *p* = 0.96): *P*_max_ = 106.0 ± 14.1%, *P*_*t*0_ = 105.4 ± 6.1%, NMDAR-STP = 65.7 ± 5.2%, *τ* = 9.3 ± 2.1 min, LTP = 39.7 ± 4.9%. The disrupted NMDAR-STP group (*P*_max_ − *P*_*t*0_ = 60.3 ± 8.2%, *p* = 0.005): *P*_max_ = 113.2 ± 6.3%, *P*_*t*0_ = 52.9 ± 4.1%, NMDAR-STP = 12.9 ± 3.9%, LTP = 40.0 ± 1.3%. (*f*) The mGluR7 antagonist XAP044 disrupted the storage of NMDAR-STP in 3 out of 9 experiments (33%). The stored NMDAR-STP group (*P*_max_ − *P*_*t*0_ = −1.4 ± 9.0%, *p* = 0.88): *P*_max_ = 96.9 ± 10.9%, *P*_*t*0_ = 98.4 ± 8.4%, NMDAR-STP = 58.3 ± 4.8%, *τ* = 7.5 ± 1.0 min, LTP = 40.1 ± 4.1%. The disrupted NMDAR-STP group (*P*_max_ − *P*_*t*0_ = 31.9 ± 17.4%, *p* = 0.2): *P*_max_ = 88.9 ± 34.7%, *P*_*t*0_ = 57.0 ± 17.6%, NMDAR-STP = 18.5 ± 6.3%, LTP = 38.5 ± 11.9%.

Indeed, all the antagonists that were used in this study could produce a loss of NMDAR-STP, irrespective of their subtype preference, while maintenance of LTP was never affected by the compounds (figure 3).

In terms of the group I mGluRs, NMDAR-STP was disrupted in 36% of experiments that used 1 μM mGluR1-selective antagonist YM298198 (*n* = 11; figure 3*a*). Inhibition of mGluR5 receptors while using the mGluR5-selective antagonist MTEP (1 μM) could also produce disruption of NMDAR-STP (30% of total experiments, *n* = 10; figure 3*b*). Co-inhibition of mGluR1 and mGluR5 by co-application of YM298198 and MTEP disrupted NMDAR-STP in 45% of the experiments (*n* = 11; figure 3*c*). Similarly, the storage of NMDAR-STP was disrupted in 47% of total experiments (*n* = 19) when using the mGluR1-preferring concentration of LY367385 (30 μM; figure 3*d*).

Baseline synaptic transmission was not affected by any of the mGluR antagonists that were used in the experiments. The level of synaptic transmission was 3.26 ± 0.68% (not shown) of baseline after 30 min application of the combination of 1 μM YM298198 and 1 μM MTEP. Likewise, the transmission was 0.94 ± 0.74% (not shown) of baseline after 30 min application of 30 μM LY367385. Notably, in experiments without the gap in stimulation, 30 μM LY367385 did not inhibit the induction of NMDAR-STP and did not disrupt its decay (figure 3*d*, grey squares). Here, the antagonist was applied prior to the TBS, and kept throughout; 30 μM LY367385 had no effect on baseline synaptic transmission after the gap in stimulation in experiments without the TBS, suggesting that the disruptive effect of LY367385 is specific to the storage of NMDAR-STP (figure 3*d*, green squares).

Similarly to the group I experiments, application of the group II/III mGluR inhibitors could also disrupt NMDAR-STP. Thus, NMDAR-STP was disrupted in 44% of cases (*n* = 9; figure 3*e*) using group II/III-preferring concentration of the competitive mGluR antagonist LY341495 (1 μM). NMDAR-STP was also disrupted in 33% of experiments using the mGluR7-selective antagonist XAP044 (1 μM, *n* = 9; figure 3*f*). Neither LY341495 nor XAP044 affect baseline synaptic transmission [45,46].

Notably, we have never observed any effects on NMDAR-STP in experiments that used a lower, more mGluR2/mGluR3-preferring concentration of LY341495 (0.1 μM, *n* = 6, not shown). In these experiments, there was no significant decrement from *P*_max_ to *P*_*t*0_ (3.8 ± 11.7%, *p* = 0.76) and NMDAR-STP (63.5 ± 12.1%) declined with *τ* 13.8 ± 2.5 min to an LTP level of 46.8 ± 8.9% (*P*_max_ = 114.0 ± 14.5%, *P*_*t*0_ = 110.2 ± 12.7%). Adjusting the overall experimental numbers for the lack of effects of the low concentration of LY341495 suggests that inhibition of both group I (21 out of 51) and group II/III (7 out of 18) mGluRs can disrupt storage of NMDAR-STP with a similar likelihood of 41% versus 39% of cases, respectively.

## 4. Discussion

It is well known that mGluRs can have a role in the induction of synaptic plasticity (reviewed in [47,48]), whereas their involvement in maintaining the potentiated state of synaptic transmission is an unexpected observation. Data, which are presented in this paper, suggest that inhibition of group I mGluRs, and also group II/III mGluRs, can prevent synapses from storing NMDAR-STP during periods without synchronous synaptic activation. We do not know whether storage of NMDAR-STP requires constitutive activation of mGluRs [49–51] or whether they are activated by ambient glutamate, or glutamate that is released during spontaneous synaptic events. Our data suggest, however, that the process of NMDAR-STP storage is not passive but metabolically active in that application of mGluR antagonist can disrupt this process.

It is surprising to observe that mGluRs, activating completely different second messenger systems, are involved in maintaining the synaptic strength during the storage of NMDAR-STP. Involvement of group II mGluRs will have to be confirmed by using compounds that are more mGluR2/3 selective than LY341495, which did not disrupt NMDAR-STP at a low concentration. On the other hand, the involvement of group I and group III receptors in the storage of NMDAR-STP is highly likely. Group I and group III mGluRs involve different effector molecules (Gq and Gi, respectively) and have opposing effects on adenylyl cyclase activity [52]. The nature of such convergent involvement of different mGluRs in the storage of NMDAR-STP requires further research. Notably, reduced induction of NMDAR-STP, and working memory deficits, have been reported in the mGluR7 knockout mice while LTP was unaffected, linking the involvement of group III mGluRs to both NMDAR-STP and working memory [53–55]. On the other hand, a relationship between group I mGluRs, synaptic plasticity and memory has also been suggested (e.g. [56], for review see [47,48]). Interestingly, activation of group I mGluRs can potentiate NMDAR responses [57,58] while activation of NMDARs can regulate the decay of NMDAR-STP after its induction [4]. Such interaction between group I mGluRs and NMDARs, which might be strengthened by the induction of NMDAR-STP, will need to be researched in future studies.

As described above, NMDAR-STP was disrupted in only about 30–50% of our pharmacological experiments, dependent on the antagonist used. Such disruption of NMDAR-STP has not been observed in the control experiments of the current study and was not dependent on the animal identity. Reliable storage of STP has also been reported in the previous publications, which investigated the storage of potentiation during time periods without stimulation [4,5,12,18]. The reason for the inconsistency of the effects of mGluR antagonists is unknown and will need to be investigated further. Notably, it has been previously shown that 3-isobutyl-1-methylxanthine, a phosphodiesterase inhibitor that raises intracellular cyclic adenosine monophosphate, can occlude induction of NMDAR-STP in about 25% of cases [16]. It is, therefore, possible that several parallel second messenger cascades, competing for some common mechanism of expression of plasticity, are involved in NMDAR-STP storage, which might explain its sensitivity to inhibition by ligands that target different mGluR groups. We cannot exclude, however, the possibility that mGluRs that are mediating the storage of NMDAR-STP are composed of unconventional subunit combinations [59].

NMDAR-STP is only sometimes [4] but not always [5,29,39] induced as a uniformly decaying exponential phenomenon and has been subdivided into fast- and slow-decaying exponential components termed STP1 and STP2, respectively [5]. Induction of STP1 and STP2 relies on the activation of NMDARs composed of different subunits [5,6,8], and it is therefore possible that different types of NMDAR-STP involve activation of different types of mGluRs, a question that we could not address in the current study. To answer this question, STP1 and STP2 would need to be induced selectively, re-examining the effects of mGluR antagonists on the processes of their storage and decay.

In conclusion, and to the best of our knowledge, this is the first report that documents the involvement of glutamate receptors in the storage of NMDAR-STP and shows that inhibition of mGluRs (groups I & III, particularly) can lead to selective disruption of NMDAR-STP without affecting LTP. It supports previous evidence that NMDAR-STP is a distinct form of synaptic plasticity [2,4,5,12,16,17,39], having unique pharmacological profile [5,6] and computational capacity [18,60,61], whereas its putative physiological role in short-term memory processes, which was originally suggested by G.V. Goddard in the 1980s celebration of D.O. Hebb [28], is yet to be confirmed *in vivo*.

## Data Availability

All data and their analyses are included in this paper. Any additional information is available from the corresponding author on reasonable request.
